# Supplementary figures and images for: Personalized computational model quantifies heterogeneity in postprandial responses to oral glucose challenge
Source: PLoS Comput Biol. 2021 Mar 31;17(3):e1008852. doi: 10.1371/journal.pcbi.1008852 (PMC8011733; doi:10.1371/journal.pcbi.1008852)

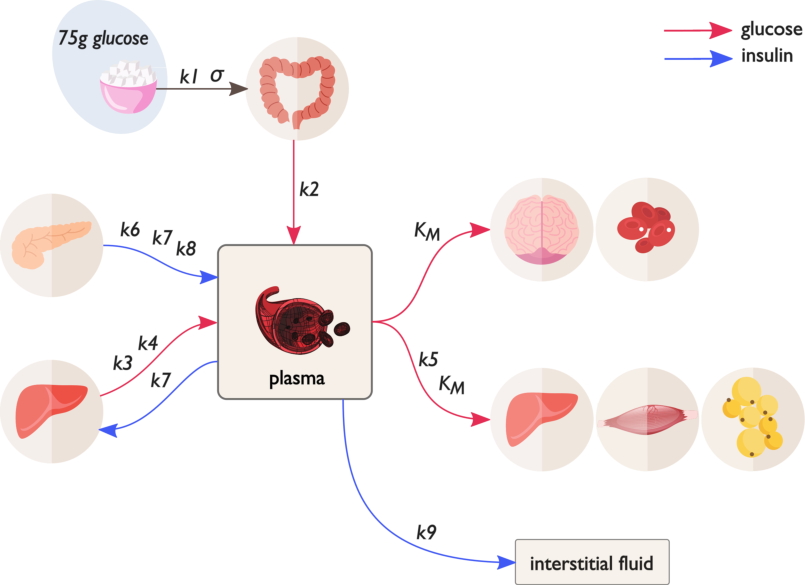

Supplement: S1 Fig — (TIF) [file pcbi.1008852.s001.tif]

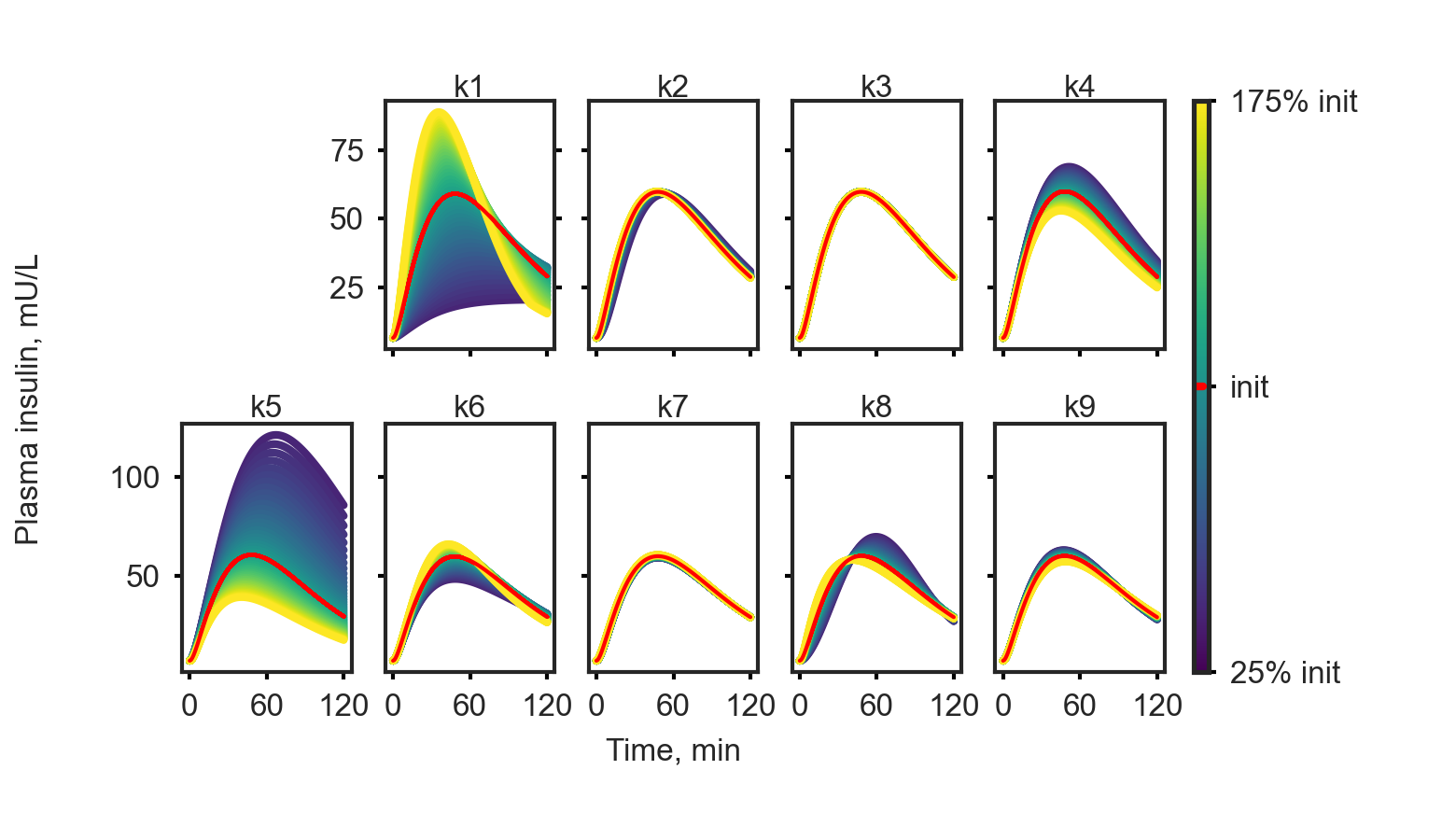

Supplement: S2 Fig — (TIFF) [file pcbi.1008852.s002.tiff]

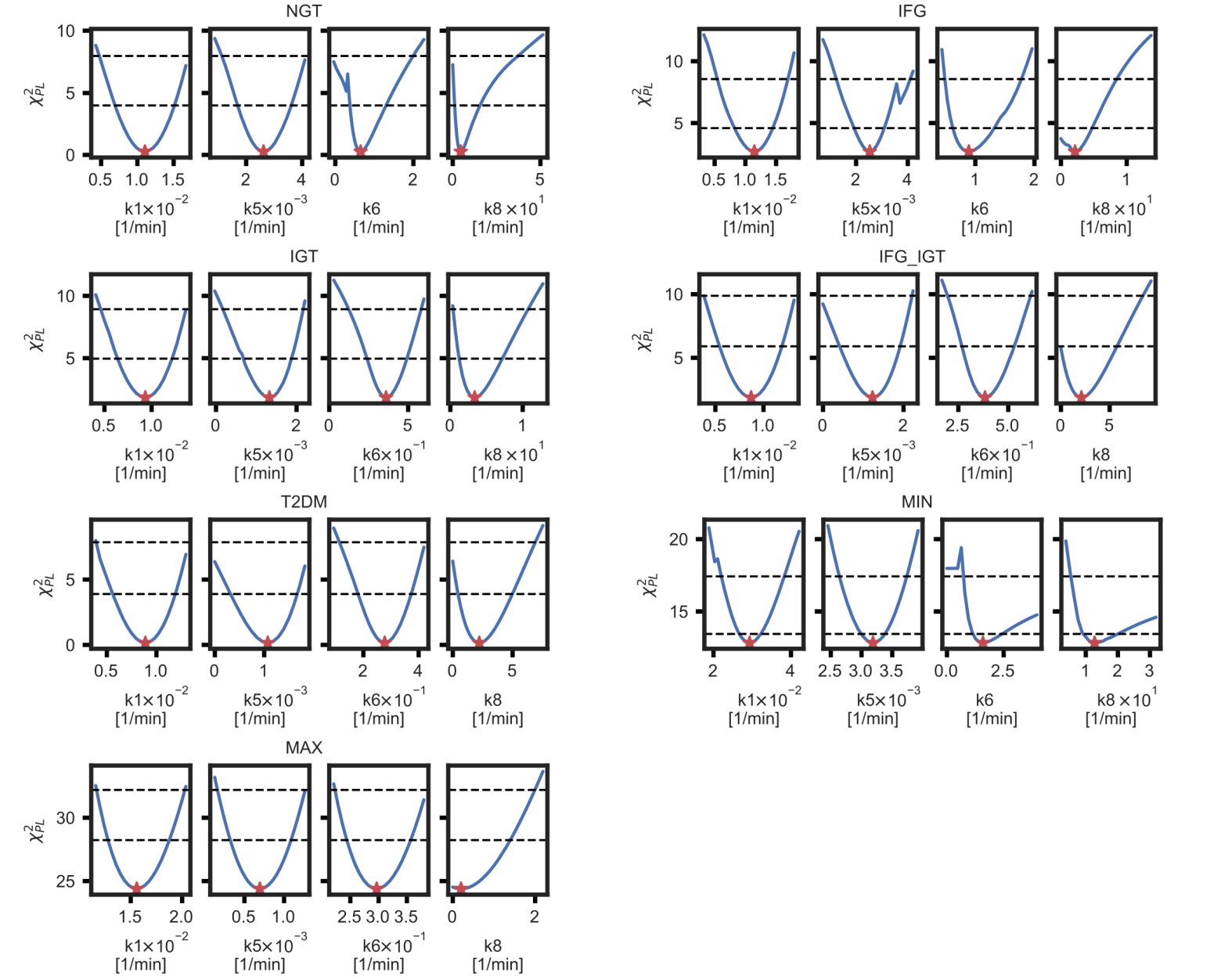

Supplement: S3 Fig — The red star indicates the SSR of the model fitted using the optimal parameter values estimated from data, while the blue line corresponds to the error as the other parameter values are being re-estimated after adjusting the parameter value iteratively. The dashed lines indicate confidence intervals where the degrees of freedom equals one (lower), and the number of parameters (upper), respectively. (TIF) [file pcbi.1008852.s003.tif]

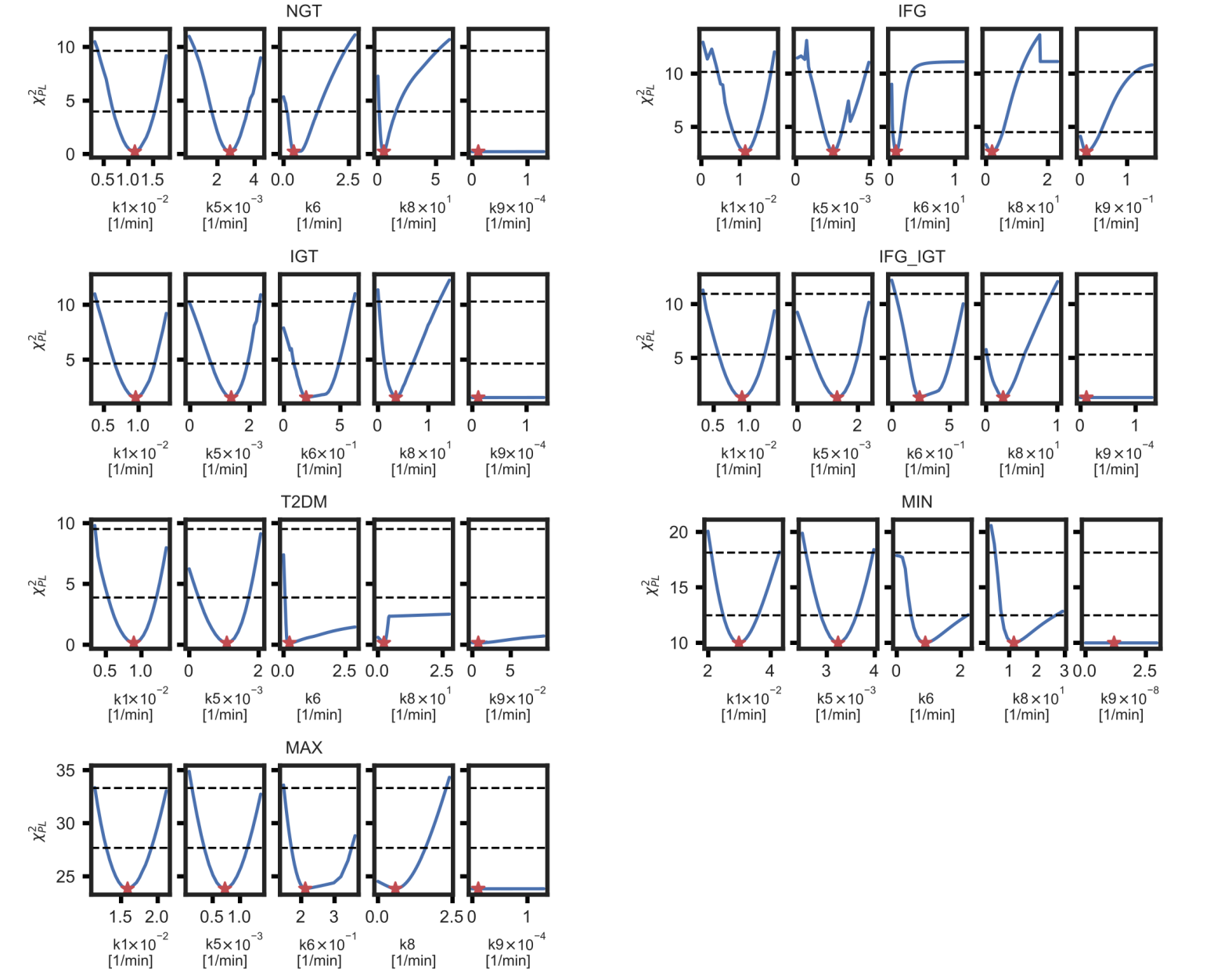

Supplement: S4 Fig — The red star indicates the SSR of the model fitted using the optimal parameter values estimated from data, while the blue line corresponds to the error as the other parameter values are being re-estimated after adjusting the parameter value iteratively. The dashed lines indicate confidence intervals where the degrees of freedom equals one (lower), and the number of parameters (upper), respectively. (TIF) [file pcbi.1008852.s004.tif]

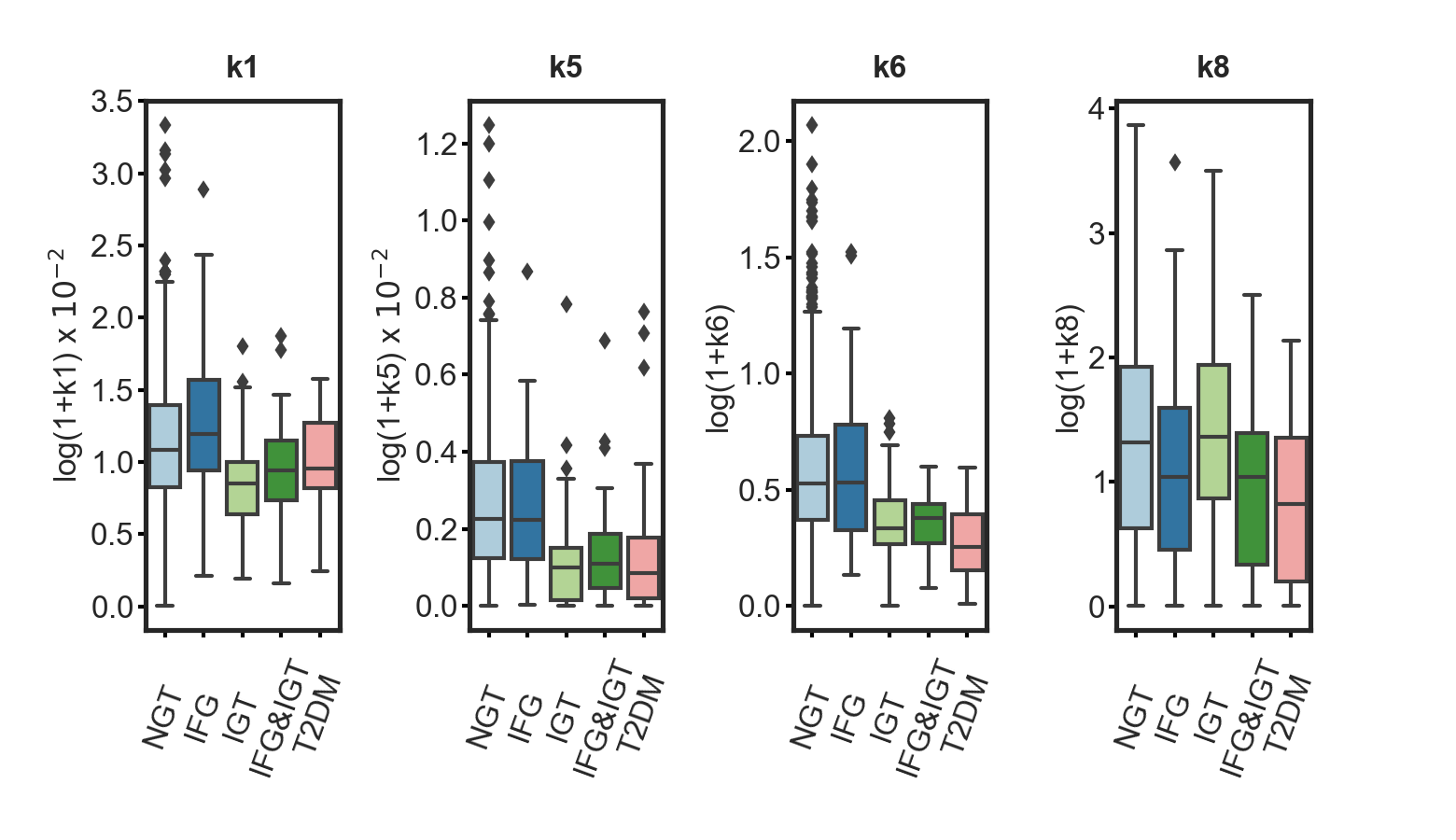

Supplement: S5 Fig — The boxes represent the 25th and 75th percentiles, the whiskers represent the min, and max values, and the horizontal line represents the median. (TIFF) [file pcbi.1008852.s005.tiff]

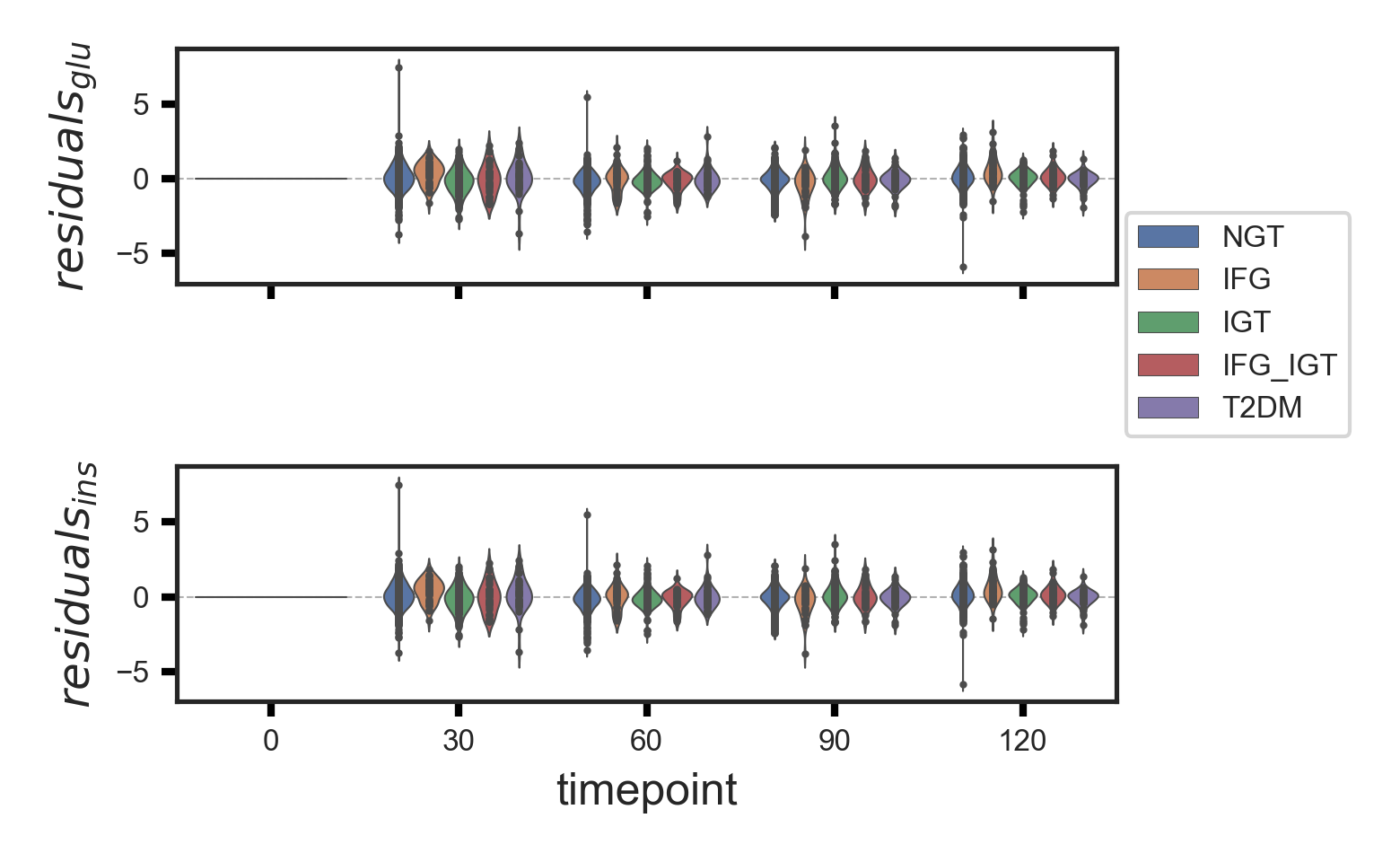

Supplement: S6 Fig — (TIFF) [file pcbi.1008852.s006.tiff]

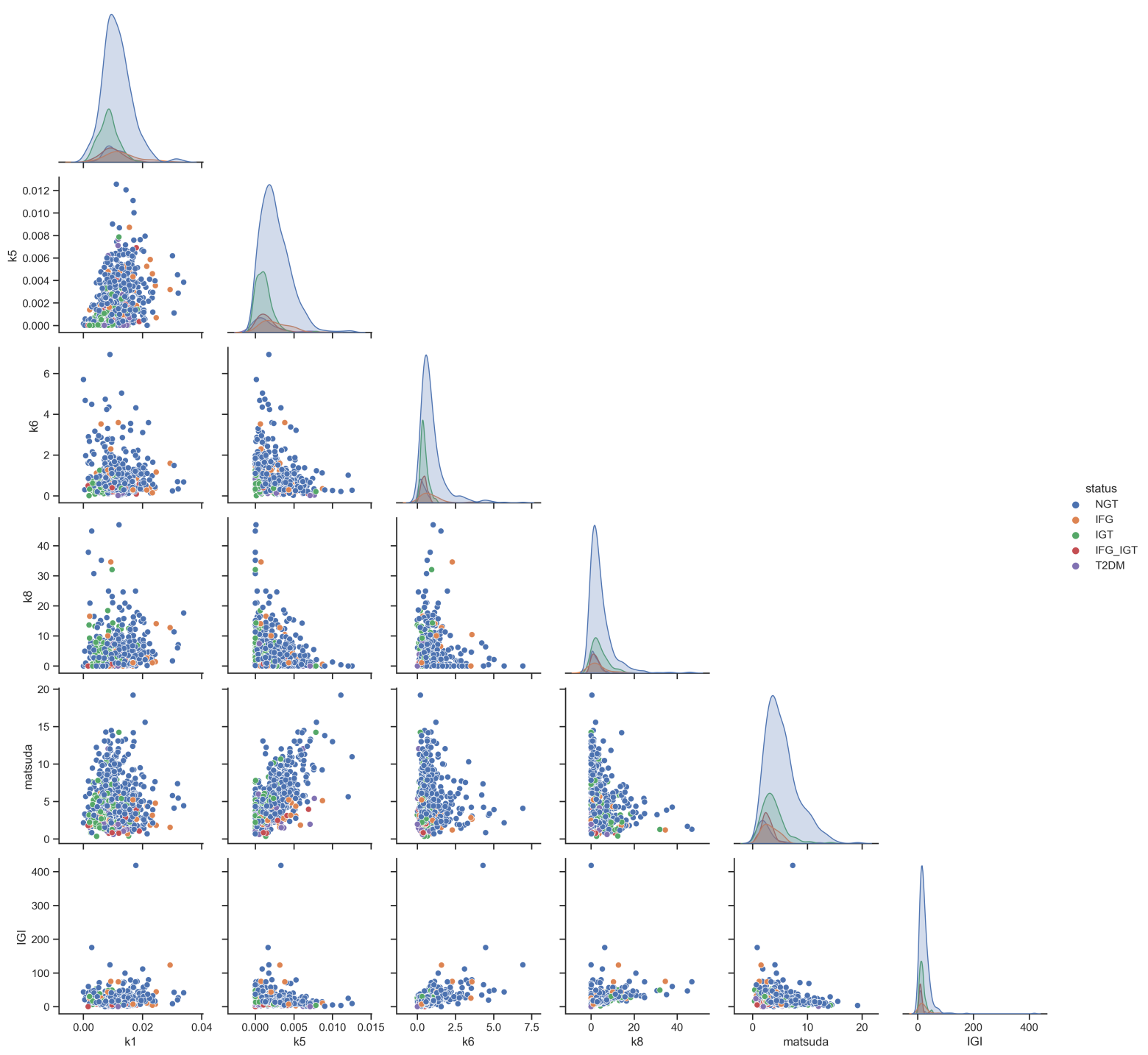

Supplement: S7 Fig — (TIF) [file pcbi.1008852.s007.tif]

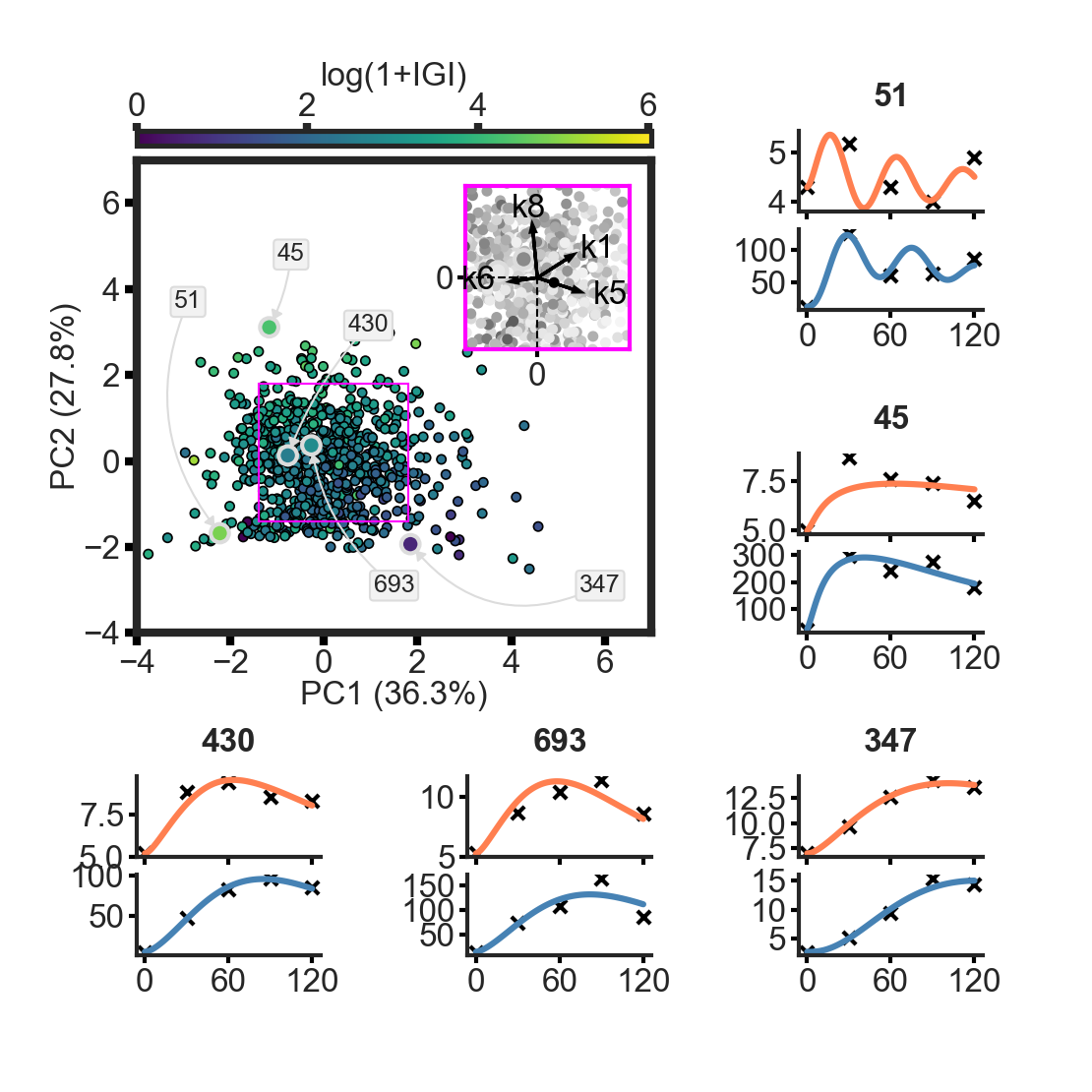

Supplement: S8 Fig — The personalized model simulations of five participants with varying first-phase insulin secretion are highlighted (individuals 51, 45, 347, 430, 693). Orange and blue lines correspond to plasma glucose and insulin model simulation, while crosses represent measured data. (TIFF) [file pcbi.1008852.s008.tiff]

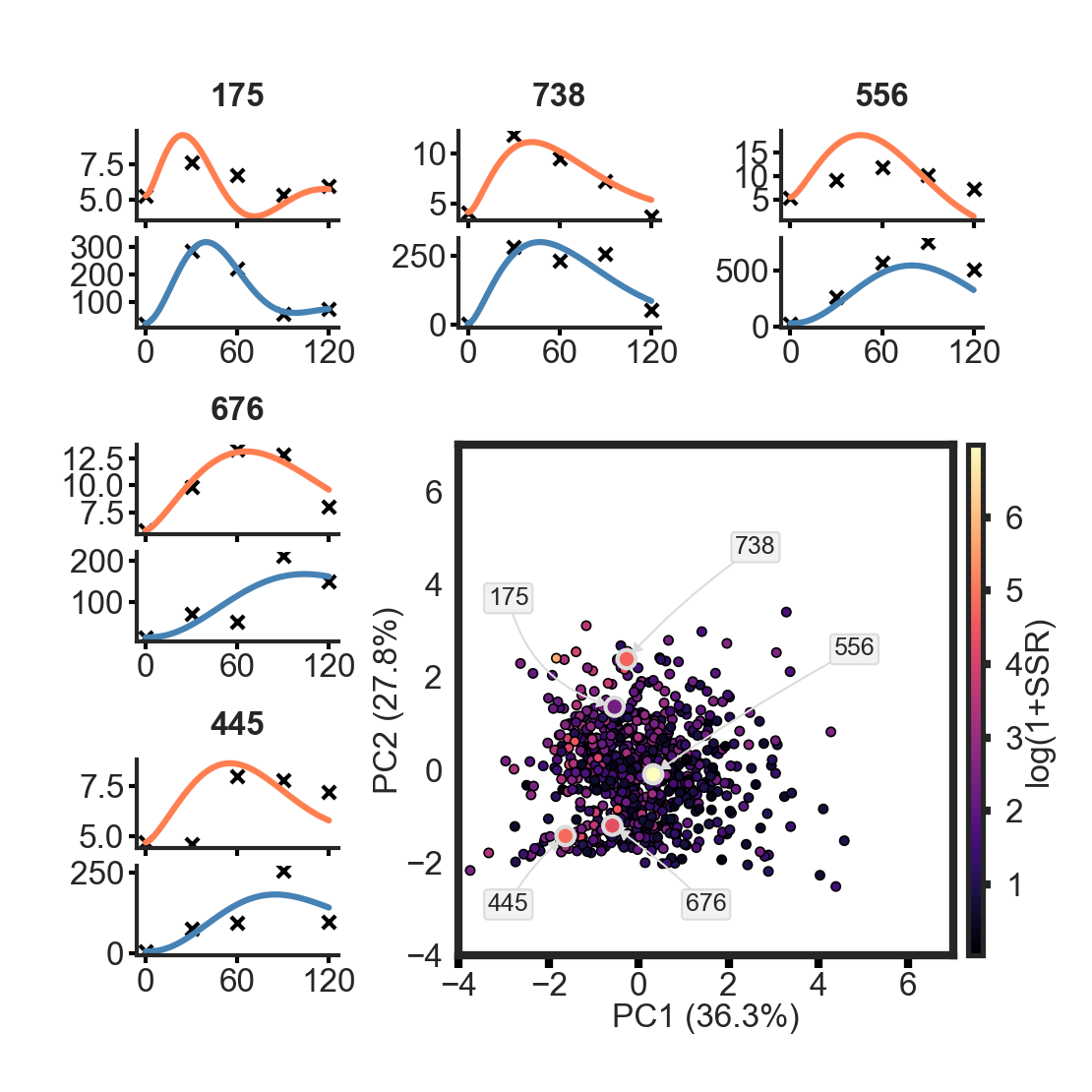

Supplement: S9 Fig — The personalized model simulations of five participants with varying first-phase insulin secretion are highlighted (individuals 175, 738, 556, 676, 445). Orange and blue lines correspond to plasma glucose and insulin model simulation, while crosses represent measured data. (TIFF) [file pcbi.1008852.s009.tiff]

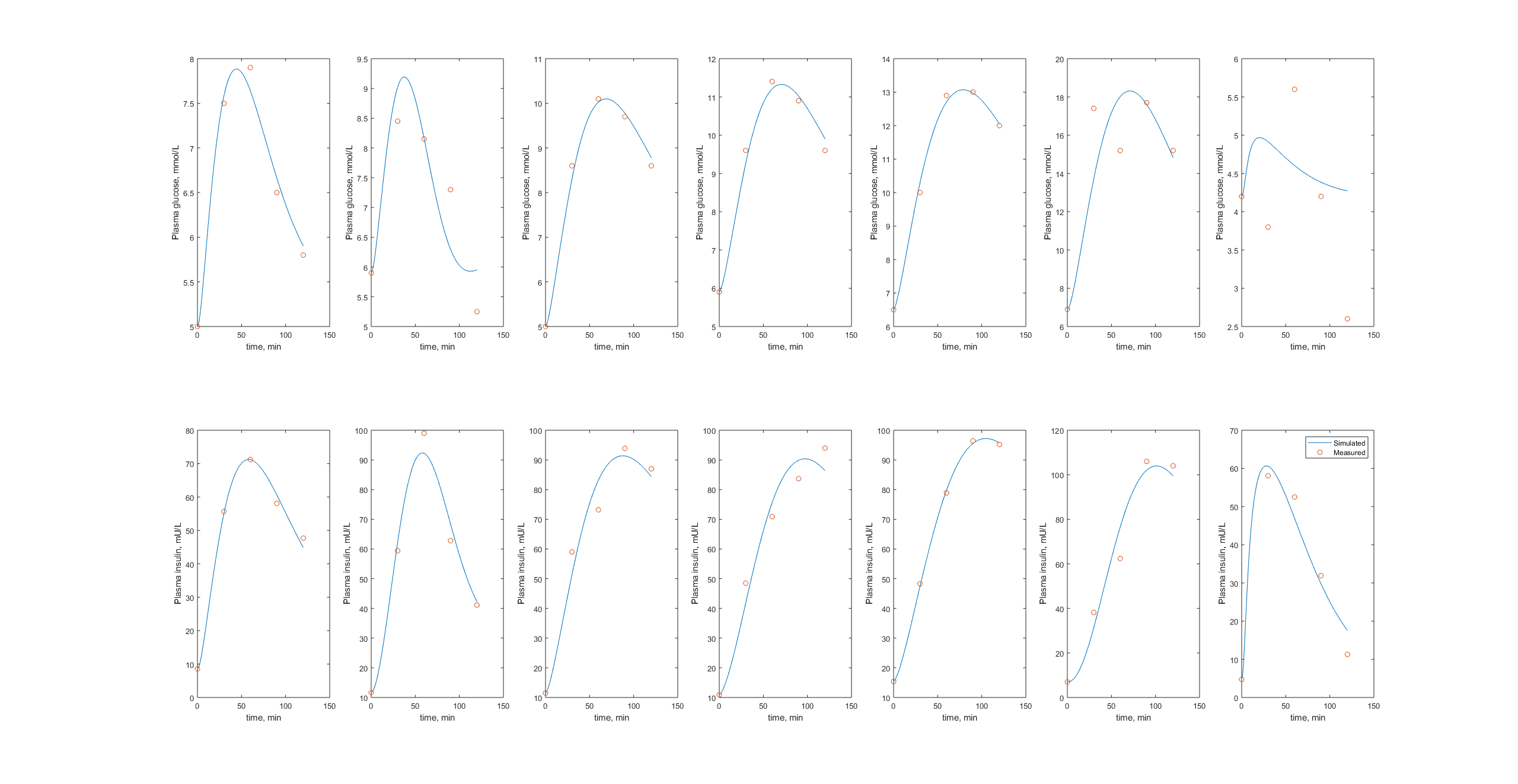

Supplement: S2 Appendix — (ZIP) [file pcbi.1008852.s011.zip › example_result.png]
