# Supplementary material for: Personalized computational model quantifies heterogeneity in postprandial responses to oral glucose challenge
Source: PLoS Comput Biol. 2021 Mar 31;17(3):e1008852. doi: 10.1371/journal.pcbi.1008852 (PMC8011733; doi:10.1371/journal.pcbi.1008852)
Supplement: S1 Appendix — (PDF) [file pcbi.1008852.s010.pdf]

S1 Appendix to Personalized computational model quantifies heterogeneity in postprandial responses to oral glucose challenge

March 8, 2021

1 E-DES Model structure

Glucose in the gut

$$\frac{dM_G^{gut}}{dt} = m_G^{meal}(D^{meal}, t) - m_G^{pl}(M_G^{gut}) \tag{1}$$

$$m_G^{meal} = \sigma k_1^{\sigma} t^{\sigma-1} exp(-(k_1 t)^{\sigma}) D^{meal} \tag{2}$$

$$m_G^{pl} = k_2 M_G^{gut} \tag{3}$$

Glucose in the plasma

$$\frac{dG^{pl}}{dt} = g^{liv}(G^{pl}, I^{pl}) + g^{gut}(M_G^{gut}) - g^{non-it}(G^{pl}) - g^{it}(G^{pl}, I^{pl}) - g^{ren}(G^{pl}) \tag{4}$$

$$g^{liv} = g_b^{liv} - k_3(G^{pl} - G_b^{pl}) - k_4\beta(I^{pl} - I_b^{pl}) \tag{5}$$

$$g^{gut} = \frac{f}{V_G M^b} m_G^{pl} = k_2 \frac{f}{V_G M^b} M_G^{gut} \tag{6}$$

$$c_2 = g_b^{liv}\left(\frac{K_M + G_b^{pl}}{G_b^{pl}}\right) - k_5 \beta I_b^{pl} \tag{7}$$

$$g^{non-it} = c_2 \frac{G^{pl}}{K_M + G^{pl}} \tag{8}$$

$$g^{it} = k_5 \beta I^{pl} \frac{G^{pl}}{K_M + G^{pl}} \tag{9}$$

$$g^{ren} = \begin{cases} \frac{c_1}{V_G M^b}(G^{pl} - G_{th}^{pl}), & \text{if } G^{pl} > G_{th}^{pl} \quad (10) \\ 0, & \text{if } G^{pl} \leq G_{th}^{pl} \quad (11) \end{cases}$$

Insulin in the plasma

$$\frac{dI^{pl}}{dt} = i^{pnc}(G^{pl}) - i^{liv}(I^{pl}) - i^{if}(I^{pl}) \tag{12}$$

$$i^{pnc} = \beta^{-1}\left(k_6(G^{pl} - G_b^{pl}) + \left(\frac{k_7}{\tau_i}\right)\int(G^{pl} - G_b^{pl})dt + \left(\frac{k_7}{\tau_i}\right)G_b^{pl} + (k_8\tau_d)\frac{dG^{pl}}{dt}\right) \tag{13}$$

$$c_3 = k_7 \frac{G_b^{pl}}{\beta \tau_i I_b^{pl}} \tag{14}$$

$$i^{liv} = c_3 I^{pl} \tag{15}$$

$$i^{if} = k_9(I^{pl} - I_b^{pl}) \tag{16}$$

2 E-DES model inputs, fluxes, parameters and constants

Table A: Overview of the E-DES model (input) variables

Name	Description	Units
t	Time	min
$M_G^{gut}(t)$	Glucose mass in the gut	mg
$G^{pl}(t)$	Plasma glucose concentration	mmol/L
$I^{pl}(t)$	Plasma insulin concentration	mU/L
D^{meal}	Glucose intake	mg
M^b	Body mass	kg

Glucose intake was set to 75g and body mass was set to 70kg.

Table B: Overview of the E-DES model fluxes

Name	Description	Units
$m_G^{meal}(t)$	Glucose mass entering from stomach	mg/min
$m_G^{pl}(t)$	Glucose mass leaving to plasma	mg/min
$g^{liv}(t)$	Glucose production by the liver (EGP)	mmol/L/min
$g^{gut}(t)$	Glucose entering from the gut	mmol/L/min
$g^{non-it}(t)$	Glucose uptake by insulin-independent tissue	mmol/L/min
$g^{it}(t)$	Glucose uptake by insulin-dependent tissue	mmol/L/min
$g^{ren}(t)$	Renal glucose elimination	mmol/L/min
$i^{pnc}(t)$	Pancreas insulin secretion	mU/L/min
$i^{if}(t)$	Insulin flowing into interstitial fluid	mU/L/min
$i^{liv}(t)$	Insulin uptake by the liver	mU/L/min

Table C: Overview of the E-DES model parameters

Name	Description	Units	Value
$k1$	Rate constant of glucose appearance in the gut	1/min	1.35e-2
$k2$	Rate constant of gut emptying	1/min	6.33e-1
$k3$	Rate constant of ΔG suppression of EGP when $G^{pl} > G_b$	1/min	5.00e-5
$k4$	Rate constant of insulin-dependent suppression of EGP	1/min	1.00e-3
$k5$	Rate constant of insulin-dependent glucose uptake	1/min	3.80e-3
$k6$	Rate constant of ΔG dependant insulin production	1/min	5.82e-1
$k7$	Rate constant of $\int G$ dependant insulin production	1/min	2.20e-2
$k8$	Rate constant of $\frac{dG}{dt}$ dependant insulin production	1/min	4.71
$k9$	Rate constant of insulin outflow from plasma to interstitial fluid	1/min	1.08e-2
σ	Shape factor	-	1.35
K_M	Michaelis-Menten constant for glucose uptake	mmol/L	0.63

Table D: Overview of the E-DES model constants

Name	Description	Units	Value
G_b^{pl}	Basal plasma glucose	mmol/L	$G^{pl}(0)$
I_b^{pl}	Basal plasma Insulin	mU/L	$I^{pl}(0)$
g_b^{liv}	Basal endogenous glucose production	mmol/L/min	0.043
G_{th}^{pl}	Renal threshold	mmol/L	9
V_G	Glucose distribution volume in plasma	L/kg	17/70
β	Unit conversion factor from glucose to insulin	(mmol/L)/(mU/L)	1
f	Unit conversion factor from mmol to mg glucose	mmol/mg	0.005551
τ_i	Integral time constant	min	31
τ_d	Derivative time constant	min	3
$c1$	Rate constant of glomerular filtration	1/min	0.1
$c2$	Rate constant of glucose uptake by the non-insulin dependent tissue	1/min	-
$c3$	Rate constant of liver insulin clearance	1/min	-

Model changes from published version

The current model has been adapted from [1]. Changes include:
1. Short and long-acting exogenous insulin inflow, c-peptide, and the parameter accounting for complex carbohydrates were removed in the model.
2. The baseline glucose (G_b) and insulin (I_b) concentrations were adjusted in model constants c2 and c3 to account for individual values.
3. Parameter k10 was removed in the model.

References

[1] Maas, A. (2017). *Playing with numbers: the development of an educational diabetes game*. Technische Universiteit Eindhoven.
