# Supplementary material for: Personalized computational model quantifies heterogeneity in postprandial responses to oral glucose challenge
Source: PLoS Comput Biol. 2021 Mar 31;17(3):e1008852. doi: 10.1371/journal.pcbi.1008852 (PMC8011733; doi:10.1371/journal.pcbi.1008852)
Supplement: S1 Table — Based on the standard two hour OGTT by the American Diabetes Association. (PDF) [file pcbi.1008852.s012.pdf]

Table S1: Criteria for prediabetes and diabetes classification based on the standard two hour OGTT by the American Diabetes Association [1]

Group[1]	fasting plasma glucose	2hr plasma glucose
IFG	5.6 - 6.9 mmol/L	-
IGT	-	7.8 - 11.0 mmol/L
IFG&IGT	5.6 - 6.9 mmol/L	7.8 - 11.0 mmol/L
T2DM	7.0 mmol/L $\leq$	11.1 mmol/L $\leq$

[1] IFG: impaired fasting glucose, IGT: impaired glucose tolerant, T2DM: type 2 diabetes mellitus

References

[1] American Diabetes Association. 2. Classification and Diagnosis of Diabetes. Diabetes Care. 2015;38(Supplement_1):S8–S16. doi:10.2337/dc15-S005.
