# Supplementary material for: Personalized computational model quantifies heterogeneity in postprandial responses to oral glucose challenge
Source: PLoS Comput Biol. 2021 Mar 31;17(3):e1008852. doi: 10.1371/journal.pcbi.1008852 (PMC8011733; doi:10.1371/journal.pcbi.1008852)
Supplement: S2 Table — Sum of squared residuals (SSR) and Akaike Information Criterion (AIC) of the ten best performing candidate models. (PDF) [file pcbi.1008852.s013.pdf]

Table S2: Sum of squared residuals (SSR) and Akaike Information Criterion (AIC) of the ten best performing candidate models

Rank	Estimated parameters	SSR	AIC
1	$k1, k5, k6, k8$	41.39	20.44
2	$k1, k5, k8, k9$	44.46	20.94
3	$k1, k5, k6, k8, k9$	37.02	21.66
4	$k1, k4, k5, k6, k8$	39.15	22.05
5	$k1, k4, k5, k8, k9$	42.57	22.64
6	$k1, k4, k5, k6, k8, k9$	35.20	23.31
7	$k1, k5, k9$	87.00	23.64
8	$k1, k5, k6$	89.75	23.86
9	$k1, k5, k6, k9$	67.94	23.91
10	$k5, k6, k8$	93.84	24.17
