# Supplementary material for: Personalized computational model quantifies heterogeneity in postprandial responses to oral glucose challenge
Source: PLoS Comput Biol. 2021 Mar 31;17(3):e1008852. doi: 10.1371/journal.pcbi.1008852 (PMC8011733; doi:10.1371/journal.pcbi.1008852)
Supplement: Attachment — Submitted filename: Response_to_Reviewers.pdf [file pcbi.1008852.s016.pdf]

# Authors' Response to Reviews of

# Individualized computational model quantifies heterogeneity in postprandial responses to oral glucose challenge

B. Erdos, B. van Sloun, M. Adriaens, S. O'Donovan, D. Langin, A. Astrup, E. Blaak, I. Arts, N. van Riel
*PLOS Computational Biology,* PCOMPBIOL-D-20-01381

---

**RC: Reviewers' Comment**, AR: Authors' Response, Line number

## Reviewer #1

**Reproducibility report has been uploaded as an attachment.**

AR: We appreciate the reviewers providing a detailed summary and criteria to facilitate reproducibility. While some of the criteria for complete repeatability cannot be met due to ethical restrictions and privacy of participant data, this should not limit others in reusing our models.

## Reviewer #2

**The manuscript by Erdos B. et al. simulates the data of oral glucose tolerance test for overweight/obese participants in DIOGene study. The authors previously launched E-DES model using already existing dataset of OGTT and in this research by fitting to individual data the weight of each parameter and its correlation to physiological findings. This model seems to be useful for estimating mechanistic insight such as individual insulin resistance or endogenous insulin secretion and evaluating the effect of interventions.**

**RC 1: In previous paper (Mass AH, et al. 2015), the parameters k5, k6, k7 and k8 were the most sensitive to change by a multiparametric sensitivity analysis. In this research, the parameter k7 is omitted on the basis of LPSA while the parameter k1 is employed to following analysis.**

**RC 1.1: What is the reason of these discrepancies between previous research and this research? Does it reflect the difference in backgrounds between previously used dataset and the data of DIOGenes study?**

AR: The model used in ref 23 (Maas et al. 2015) has a slightly different formulation to the one used in this study (details are described in S1 Appendix), which may lead to different sensitivity analysis results. However, our sensitivity analysis results are in agreement with those detailed in ref 24 (Maas et al. 2017), where k1 was found to be sensitive and k7 was found to be insensitive by both LPSA and MPSA. Furthermore, in Maas et al. 2015 the authors selected k5, k6, k7 and k8 because they hypothesized that the difference in responses between their studied populations should originate in differences in insulin secretion and insulin sensitivity, processes captured by these four parameters. We adopted a slightly different approach where we allowed k1-k9 to be selected based on a data driven fitting scheme, rather than utilizing prior hypothesis on which of these parameters to select.

**RC 1.2: The parameter k1 changes the peak concentration and time of gut glucose mass resulting in plasma glucose concentration. The authors should discuss about the association between the factor regulating parameter k1 (e.g. intestinal motility) and the variation of overweight / obesity subjects.**

AR: The parameter k1 represents the rate of glucose appearance in the gut, indeed including processes such as gastric emptying and intestinal motility. We agree with the reviewer that there may be an association between the processes regulating k1 and BMI. However, in our tests no association was found between the parameter estimates of k1 and BMI as well as no difference between the parameter estimates in overweight compared to obese subjects. We have included these remarks in the discussion on `page 20, lines 411-413`.

**RC 1.3: Is the parameter k1 also higher level in NGT or IFG than ones in IGT, IFG&IGT and T2DM? As well as the parameters k5, k6 and k8 (line 250-254), this should be mentioned.**

AR: Indeed the estimates for parameter k1 also tend to be higher on average in the NGT and IFG groups compared to the IGT, IFG&IGT and T2DM groups. This has been corrected on `page 13, line 269-270`.

**RC 2: Although the authors classify the participant in DIOGenes according to ADA diagnosis criteria (line 144-147), the median of blood glucose in figure 2 seems strange. Since there may be some errors in column annotations, please explain with actual median values and correct if needed. When corrected, please mention about the rightfulness of following analysis.**

- **Plasma glucose level at 120 min in IGT seems lower than 140mg/dL.**
- **Plasma glucose level at 120 min in IFG seems higher than 140mg/dL.**
- **Plasma glucose levels at 0 min in IGT and IGF are higher than one in IFG&IGT.**

AR: We are grateful for the reviewer for pointing out our mistake here. The data was used in the wrong order causing a mismatch of the subplot headers and plotted data. The plots have been rearranged to their correct subplot header. To further clarify, the ADA diagnosis criteria used were also added to the supplementary material Table S1.

**RC 3: Can this model predict the OGTT patterns of new subjects? In other words, are these significant parameters of k1, k5, k6 and k8 able to be predicted by some other physical parameters (e.g. Body mass index, HOMA-IR / Matsuda index, HbA1c, etc)?**

AR: In order to predict the OGTT pattern of new subjects the model parameters must be specified. The most accurate simulation results from the parameter set that was estimated on that individuals' data. However, reasonably accurate simulations may be made by only using an individuals' fasting glucose and insulin measurements with a parameter set pertaining to a relevant population.

We did not find any association between the model parameters with BMI, HOMA-IR, HOMA-B, HbA1c. We report a moderate association between the parameter k5 and the Matsuda index as well as a moderate association between the parameters k6 and k8 with the insulinogenic index. The lack of association between parameters with BMI, HOMA-IR and HbA1c was expected, as these physiological measures represent steady state and overall glycemia and fail to capture the short-term postprandial dynamics. In conclusion, the parameter estimates cannot be predicted by other simpler physiological measures. We advocate our approach as a tool that is capable to derive

more detailed/nuanced information from the standard 5 time-point OGTT as we discuss on page 20, lines 420-427.

**RC 4:** **PCA analysis for these four parameters shown in figure 5 is very interesting. At a glance, NGT is a most heterogeneous population but no significant difference between groups (e.g. no group make clear cluster or tendency).**

**RC 4.1:** **In other words, no differential tendency of diagnostic classification along with vectors of the parameter k1, k5, k6 and k8 is shown.**

**RC 4.2:** **In terms of the pathogenesis of diabetes, T2DM will show lower insulin secretion (k6 and k8) and/or insulin resistance (k5). How the authors explain this discrepancy? The opposite direction of k5 and one of k6 or k8 may be explanation for it, is it a feature of this modeling or used data set?**

AR: (4.1) The development of T2DM happens continuously over a prolonged period of time: as insulin resistance progresses, insulin secretion first compensates and later declines due to prolonged stress on the pancreatic beta islets. Subjects' responses here represent snapshots throughout this trajectory. Therefore, we believe that the lack of clustering and more of a spectrum along the loading directions is sensible. Furthermore, the ADA criteria while also measured from the OGTT, capture a different property than what our personalized models do. The ADA thresholds are sufficient for clinical diagnosis of prediabetes and diabetes, however they fail to capture the nuances in the responses that may ultimately yield therapeutic implications.

(4.2) It is important to note that the Diogenes study aimed at recruiting a reasonably homogeneous population of overweight/obese but otherwise healthy individuals. As the reviewer suggests the discrepancy may be a result of the study sample (40 T2DM, 496 NGT). In addition, it may also be a result of how the ADA criteria are defined. We argue these points on page 19-20, lines 395-406. The parameter k5 represents insulin sensitivity, therefore we believe that it being lower in T2DM (as well as IGT and IFG&IGT compared to NGT and IFG) is logical (Figure 5).

**RC 5:** **In figure S5, the association between SSR and the four parameters are well described. Participant 175 shows higher SSR resulting incorrect simulation for the parameters. While participant 556 shows notably high SSR but the parameters locates within an average range. This may be a result of the difference in absolute value of plasma glucose concentration. The authors may be better to normalize the SSR using absolute value.**

AR: Indeed, the interpretation of SSR is difficult in this situation. We agree that a form of scaling should be included in order to disentangle the fitting of the shape from the absolute values. Unfortunately, we ran into considerable difficulties in norming by absolute value due to the time-series property of our data. The choice for norming (e.g. fasting absolute value or mean abs value or maximum of the time points) all lead to different forms of bias. Therefore, we decided to keep the raw SSR in the plot and elaborate its caveats (page 18-19, lines 371-373). We are considering developing a more appropriate error function in the fitting of the glucose and insulin values that may alleviate some of the issues in follow-up work.

**RC 6:** **The parameters visualized to reflect the physical status are most important and interesting in this research.**

**RC 6.1:** **The authors visualized the correlations between these parameters and insulin sensitivity in figure 5. While endogenous insulin secretion from pancreatic beta cells is also important on**

**the pathogenesis of type 2 diabetes. Insulinogenic index calculated from OGTT result is often good indicator of endogenous insulin secretion. The association between these parameters and the indicator of endogenous insulin secretion should be shown.**

**RC 6.2: In this study, can any alteration in these parameters after intervention be observed?**

AR: (6.1) The authors are grateful for the suggestion of including the IGI as an indicator of first phase insulin secretion. An additional figure (Fig S8) has been added to the supplementary material showing the parameter space of the model colored by the insulinogenic index and highlighting some examples of varying levels of insulin secretion. The model parameters k6 and k8 show an association (r=0.56, r=0.49 both p<0.001 respectively) with IGI. The results are further elaborated on `page 13-15, lines 274-280` and `page 20, lines 416-423`.

(6.2) Studying the intervention effect using the model was out of scope for this study. However, indicating changes in the insulin regulated glucose homeostasis via the parameters of the fitted model is one of the key features of this approach, and its application to the intervention effect should be examined in future work.

## Reviewer #3

**In this manuscript, the authors fitted their previous well-established physiology-based dynamics model of glucose-insulin system to individuals' data to elucidate the heterogeneity in individuals' responses. In order to reduce the number of estimated parameters for individualized models, the authors did sensitivity analysis, model selection based on Akaike information criterion (AIC), examined the parameter identifiability using profile likelihood, and finally chose the model with four parameters (k1, k5, k6, k8) to be estimated. The individualized model fitted pretty good, although the authors also reported its inability in some cases. Based on the estimated parameters of individualized models, the authors also did principal component analysis (PCA) and showed correlation between k5 (rate constant of insulin-dependent glucose uptake) with Matsuda index.**

**Overall, this is a pretty good study. The manuscript looks quite clear and easy for understanding. The purpose of this study developing individualized models seems to elucidate the heterogeneity in individuals' responses. It might be better if the authors could provide some more details in the application of these individualized models. And I listed some other comments as follows.**

**Major comments:**

**RC 1: Page 10/22 Line 200, 'inability to go below fasting value', is it possible that this behavior is due to magnitude difference in k10 and k3 (as shown in Appendix S1 Table 3). In fact, I did not find k10 = 2.60 in ref [23]; I am not sure if I missed something. With similar thoughts, since these rate constants k1-k10 are all multiplicative in the dynamics equations, maybe the authors should explore some magnitude of possible values (such as 0.01X, 0.02X, 0.05X, 0.1X, ... to 100X) instead of current explred range 25% to 175%? And maybe that is part of the reason why some of the rate constants are not sensitive (as shown in Fig 1).**

AR: We are grateful for the reviewer pointing out the missing reference to k10=2.60, indeed this value has been adopted from unpublished work. After considering the arguments pointed out in [Maas AH, 2017; ref 24 previously 23], it seems that this parameter is generally hard to estimate, and

remains unidentifiable in most cases. Considering the effect it introduces in the simulations (inability to go below basal glucose, as mentioned above), we have decided to remove k10 altogether, thus reverting to the model that has parameters k3, and k4 controlling endogenous glucose production (EGP) in the model. This part of the model (EGP) is also equivalent to the one reported in the original publication [Maas et al 2015, ref 23]. The advantage is that the simulations are now able to go below basal glucose in a continuous fashion. However, the previous formulation of EGP prevented the model from entering an oscillatory state that sometimes arises with responses going below basal levels (see example Fig 4-C individual 129 or Fig S8 individual 51). We concluded that the formulation that allows the simulations to go below basal levels is a better representation of glucose homeostasis even though the potential erroneous oscillatory simulation in a few cases. We have adapted the text on page 10 line 200 (`page 11, line 218` in the revised manuscript). We have removed the discussion pertaining to the inability of the model to go below basal glucose, and included some discussion on the oscillation issue at `page 19, line 386-390`. However, introducing further modifications to the model is outside of the scope of the current study.

With regards to the magnitude choice in the sensitivity analysis, larger magnitudes have been explored in [Maas AH, 2017; ref 24 previously 23], concluding that after a given point the model goes into unwanted oscillation. Therefore, we only used a limited range that has been validated to be able to capture the responses in such a population as in our study without the unwanted behaviour.

**RC 2: Page 12/22 Line 255, 'significant positive correlation with the Matsuda index'. Could the authors show a scatter plot between the two variables for the reported correlation?**

**Similarly, for better understanding of the heterogeneity, it might be better to also show the pairwise scatter plot with density contour between k1, k5, k6 and k8, in additional to the boxplot in Fig 4 and the PCA plot in Fig 5.**

AR: An additional figure showing the requested scatter and density plots (Fig S7) has been added to the supplementary material and reference to the plots were added to the text on `pages 13, 15, lines 277 and 280`, respectively.

**RC 3: Page 14/22 Line 318, 'as this type of response is rare in modelled population'. How many cases are with this type of response? Will it help if the authors first select out these cases, and then estimate the parameters on this group of people?**

AR: There were in total 66 (9% of all responses; added to `page 17, line 326`) such cases in this data set. Indeed, we have tested the pipeline separately for these cases, hoping that a different combination of parameters would yield better results. However, what we found was that while estimating more parameters lead to simulations more in agreement with data, the parameters were very rarely identifiable. Therefore, any conclusion on the parameter values would be misleading. We believe that potential remedies to this issue would require extensive changes to the model, which is outside of the scope of the current study.

**RC 4: Page 15/22 Line 335, 'The individualized models showed a 4-5 fold derease in SSR in all groups compared to group simulations, indicating that the model individualization was successful'. I am wondering the individualized model is estimating 4 parameters to fit 8 data points, right? It might be expected that individualized models would have smaller SSR. Although the authors have shown some cases of under-fitting (Fig 3-B, 3-C, and Fig S5), is it possible for the individualized models over-fitting the data? In other words, how reliable are the estimated parameters of individualized models? If it is estimating 4 parameters to fit 8 data points, and over-fitting**

**might be an issue, then two possible suggestions might be: (1) using Bayesian framework on parameter estimation; (2) exploring the relationship between individuals by similarity in their data points (e.g. hierarchical clustering or tSNE embedding), and then estimating and possibly smoothing the paramters considring the group of neighbors.**

AR: Indeed, it was expected that the individualized models would have a smaller SSR. However, as the responses on the individual level take on more complex shapes than the population median responses, we think that it is not trivial that the individual models consistently show a lower SSR compared to the population models. In our approach we try to find a balance between under- and overfitting by implementing a penalty via the Akaike Information Criterion (AIC) on the number of parameters used in the model. The model selected this way (with parameters k1, k5, k6, k8) is deemed optimal by the AIC (SSR=41.39; AIC=20.44). A model with more parameters fits the data only slightly better (k1, k5, k6, k8, k9, SSR= 37.02;AIC=21.66) while a model with less parameters (k1 k6, k9, SSR=87.00; AIC=23.64) fits it much worse. Furthermore, after the selection process, parameter identifiability was also assessed via profile likelihood analysis to assess whether the parameter values are unique in a tested range. However, these analyses were carried out on the group median and extreme responses and not on every individual model. Therefore, as the reviewer points out, in individual cases, the model might still show under/overfitting.

One could carry out the whole approach on every individual curve and find an optimal model. However this may result in a different parameter set for every individual, which would prevent the comparison of the models. We hope that our approach strikes a good balance in capturing the variation in the population while still maintaining the important property to compare individuals with each other.

The authors are grateful for the suggestion of the Bayesian parameter estimation framework and the more elaborate clustering analysis to allow for better groupings and consequently parameter estimates. Our aim was to estimate individualized model parameters with a focus on fitting the individuals' response. We believe that Bayesian or mixed-modeling approaches would yield more conservative parameter values in the case of individualized models, constraining them to be closer to the population values. While this might be very beneficial in some cases (e.g. preventing the simulation of outlying responses), it was not the focus in this study. However, we will keep these recommendations in mind for further studies.

**RC 5: Fig 3B, participant 556, it looks weird to see a sharp angle on the right of the red curve when it reaches the fasting level, which might indicate that the parameters for glucose level less than its fasting level are wrongly specified in magnitude difference, which might be the reason for the inability of the model going below fasting value.**

AR: We appreciate this insight, it is indeed the issue with the magnitude difference between the parameters k3 and k10. As mentioned in our answer to comment 1, we removed the parameter k10, therefore reverting to the original description of endogenous glucose production (EGP) described in [23] and circumventing the issue of the sharp angles and inability to go below basal.

**Minor comments:**

**RC 1: Page 6/22 Line 118, 'a weight of 0.1', is the weight inside or outside the square of formula (1)? And m and N in formula (1) might be better defined or explained. Instead of fitting to median (or min or max) responses, is it possible or better to estimate the parameters with confidence interval of the model by fitting it to all the data for each group (NGT, IGF, IGT, IGF&IGT,**

**and T2DM)?**

AR: The manuscript has been adapted on page 7, line 123-127 to include the suggested correction regarding the formula definition and the weighting factor. The suggested parameter estimation technique requires either a Bayesian or mixed modelling framework that was outside the scope of this study, please see our elaborated answer under major comment 4.

**RC 2: Page 7/22 Line 132, 'original publication' might be better have the citation to ref [23].**

AR: The manuscript has been adapted on page 7, lines 121-122 to include the suggested improvement.

**RC 3: Page 8/22 Line 152, 'seven curves' looks unclear and confuses me, until I saw Fig 2.**

AR: The text has been adapted to clarify this inconsistency on page 8, line 162-163.

**RC 4: Page 8/22 Line 161, 'X^2(alpha, df)', what number are used as alpha and df? And this term 'X^2(alpha, df)' is not shown exactly the same in formula (2).**

AR: The manuscript has been adapted on page 9, line 171 to correct the mistake. We added clarification on the alpha and df on page 9, lines 173-175.

**RC 5: Page 11/22 Line 241, 'the model could replicate accurately (e.g. participants 110, 513, Fig 3-C).' The fitting to data points of participant 110 looks not so accurate, while the fitting to data points of participant 513 looks much more accurate.**

AR: The manuscript has been adapted on page 13, line 260 to include the suggested improvement.

**RC 6: Fig 4 legend, box-plot elements might be better defined, such as median, box limits, and whiskers. And 'Fig S3' in Fig 4 legend might be typo of 'Fig S4'.**

AR: The figure 4 (figure 5 in the revised manuscript) legend has been adapted to include the suggested improvement, and the label mistake has been fixed.

**RC 7: Page 16/22 Line 367, 'individuals ... due to unually high insulin values still end up in the NGT group'. How many such cases in 496 NGT individuals?**

AR: While this is a very interesting and important point especially from the modelling perspective, it is difficult to answer due to a lack of clinical or biological definition on what is considered to be high insulin levels. We believe that our model may help pinpoint such responses as we elaborate on page 19-20, line 402-410.

**RC 8: Fig 1, the label of y-axis might be better as 'Plasma glucose, mmol/L'; or mention it in figure legend.**

AR: The figure 1 (figure 2 in the revised manuscript) has been adapted to include the suggestion.

**RC 9: Fig 5A-B, the color coding might be too close for distinguishing (e.g. IGT vs IFG); and for matsuda index, it might be better colored in monocolor, e.g. white-green-black.**

AR: We have considered the monocolor option as it would also be clearer that the coloring depicts a single scale, however in our opinion it was even harder to distinguish similar cases using that color scheme. Therefore, we resorted to the 'viridis' color-scheme due to its documented property of easy readability also in the case of color blindness.

**RC 10: Fig 5B, The loading vector subplot is not mentioned in figure legend; and no explanation of**

**the color of the gray scatter points in that subplot.**

AR: The figure 5 (figure 6 in the revised manuscript) legend has been adapted to clarify that the loading vector subplot is an insert.

**RC 11: Table S2, it might be better if the authors could show some percentiles (e.g. 5%, 25%, 50%, 75%, 95%) in addition to the mean and standard deviation of each parameter. And since the parameters are multiplicative, and Fig 4 shows near normal after log-transformation, the standard deviation without log-transformation might not be very meaningful.**

**In addition, it might be helpful if the authors could compare Table S2 with the estimates with confidence intervals of the models fitted to the median responses.'**

AR: Indeed, the Table S2 is not very informative. We have added a figure (Fig S7, referenced in the text on `pages 14, 15 and lines 277 and 280`, respectively) depicting the parameter distributions on their original scale and removed the redundant Table S2. We chose to avoid statistical comparison of the parameter estimates on the various population median responses, due to the large heterogeneity observed on the individual level in each group. Our goal in estimating the individual parameters was to highlight heterogeneity within each group. We advocate the evaluation of the whole set of parameter values estimated for an individual as we try to elaborate with figures 6, S8 and S9.

## Reviewer #4

**Erdos et. al. present a model aiming to better characterize the heterogeneity in individual responses to oral glucose challenges. The authors build "personalized models" for each individual by fitting a subset of parameters to each individual. Overall, I have no explicit concerns with the technical aspects of the work. However, the authors could do more to make the manuscript accessible to readers, to place their work in context of other nonlinear mixed effect modeling approaches, and to bridge towards how this work can be clinically translated.**

**Specific points:**

**RC 1: When the authors use words like "individualized models" and "model selection," this generally implies that different model structures are being compared, or selected for each subject. I'd suggest wording like "personalized models" and "selection of parameters for personalized fitting" to make it clearer that all individuals get the same model structure but personal parameterizations.**

AR: We appreciate the reviewer pointing out the inconsistency and also providing appropriate improvements. The manuscript has been adapted to consistently use "personalized models" throughout the manuscript as well as in the title. We believe that selecting which parameters to estimate and which ones to fix has a large effect on the flexibility of the model and in this sense changes the model structure. In addition to this, we prefer to keep the term "model selection" for it's brevity as well. However, the relevant methods section has been revised to clarify the approach, indicating that indeed the same set of parameters are estimated in personalized models. (`lines 128-140, 150-163, 201, 308-313`)

**RC 2: While Appendix S1 provides details on the model, it would be very helpful to include a figure schematic in the main text for readers to reference. For example, when looking at the list**

**of parameters to be individualized, this would help the reading think about what processes k1, k4, k5, k6, k8, and k9 represent, compared to the processes for which parameters are not individualized.**

AR: We appreciate the suggestion, we agree that including a schematic makes the manuscript more self contained. Therefore, we have included a schematic of the model in the supplementary material (Fig S1) as requested. This is now referenced in the text on `page 6, lines 95-96`.

**RC 3: Dmeal (food intake): Clarify in Table S1 of appendix S1 that this is specifically glucose (since an OGTT, not all food intake).**

AR: The requested specification has been added as a footnote to the Table S1 (Table S2 in the revised supplement).

**RC 4: Given the population and individualization steps, and then the subgroups considered, it would be helpful to the readers to provide a flow chart of the model building process. From just the text, it is a bit hard to clearly understand the full process.**

AR: A schematic of the approach (Fig 1) for obtaining the parameter values to estimate in individual specific models has been added to the manuscript as suggested. Furthermore, the relevant methods section has been revised to clarify the steps. (`lines 128-140, 150-163, 219-221, 234`)

**RC 5: In the methods or supplement, please lay out the ADA diagnosis criteria, for reference by unfamiliar readers.**

AR: As suggested, a table (Table S1) detailing the ADA diagnosis criteria was added to the supplement.

**RC 6: Parameter estimation step: was this fit to all data (naïve pooled) or mean values for the population?**

AR: The parameter estimation procedure as outlined in section "Parameter Estimation" works on single time series (e.g. median values in case of a population response and the individual values in case of individual responses). In part (ii) of the parameter selection process, the model was fit on the "representative" cases (NGT, IFG, IGT, IFG&IGT, Min, Max) which are the median values per ADA group plus two individual responses. Clarifications were included on `page 8, lines 153-157`.

**RC 7: Model Individualization Pipeline: Why were non-individualized parameters set to literature values instead of the population values fit in the parameter estimation step?**

AR: The reference parameter values in the original publication have been determined with a more extensive parameter search procedure across multiple data sets that are similar to the one used in this study. Therefore, we opted to use them as population reference values. Furthermore, estimating all parameters from 10 time-points (using our parameter estimation approach) is still problematic in terms of reliability. Consequently we decided to fix the non-individualised parameters to the values presented in the original model publication. However, the suggested approach may be beneficial, especially if the suggested mixed modeling framework or a Bayesian framework is used to estimate the population parameters (please see our answer @ Reviewer 3 comment 4, Reviewer 4 comment 8).

**RC 8: Can you discuss why you chose this approach over a "traditional" nonlinear mixed-effect modeling approach, which also lets you fit individual estimates for some parameters and fix other to population levels? I am not suggesting the authors change their methodology, as both approaches are reasonable, but I think this is worth discussing; a fair bit of the modeling to**

**date on insulin-glucose dynamics (e.g. IGI model, which should also be referenced), and many members of the authors' prospective audience here come from the pharmacology field, where many would expect to see this done in a nonlinear mixed-effect modeling approach. This is particularly relevant as the authors present this as a generalizable pipeline.**

AR: Our choice to use a standard maximum likelihood estimation to estimate the parameters was mostly based on convenience, as we've been using this approach extensively. We believe that our approach to go from a population model to an individual specific model is generally the same irrespective of the parameter estimation technique. The steps of fixing the values of insensitive parameters and further reducing the number of parameters by considering model fit and parsimony would largely remain the same no matter the parameter estimation technique. However, we fully acknowledge the validity of the other approaches suggested by the reviewer. We have included some remarks to this in the discussion on page 18, lines 359-366.

We have included some remarks about the IGI model as suggested in the introduction (on page 4, lines 48-53).

**RC 8.1: The authors do compare individual and population performance by looking at each of the patient subgroups. This verification is reassuring (It would be concerning if the individual models to not do better than population), and Figure 3, laying out some of the interesting individual fits and patterns is quite nice.**

**RC 8.2: However, the authors could go farther in diagnosing individual and population model predictions, as well as visualizing distributions of individual parameters and residual errors. This would allow readers to get a feel for how well the model is performing, and whether certain assumptions one generally makes with this sort of modeling are being met. Figures 6 and 7 in this tutorial provide a good example of the type of diagnostic plots that would be helpful. They don't need to be exactly the same (e.g. I don't expect conditional weighted versions of residuals), but generally would help show if the individual and population components of the model are suitable and well-behaved: https://ascpt.onlinelibrary.wiley.com/doi/full/10.1038/psp.2013.14**

AR: Indeed, as the reviewer points out, the individual models showing lower error than the population ones is not surprising, however, as the responses on the individual level take on more complex shapes than the population median responses, we think that it is not trivial that the individual models consistently show a lower SSR compared to the population models. We appreciate the recommendation to use the diagnostic plots as seen in Fig 6 and 7 in the referenced material. We are not sure if these exact diagnostics are applicable to our case, since in these figures, the residuals originate from one model, whereas we estimate a separate model for every individual. However, to elaborate on the goodness of fit as suggested, we have included an additional figure (Fig S6) in the supplementary material showing the distribution of the residuals per time-point, pooled across the individual models, which is also referenced in the main text on page 19, line 371.

**RC 8.3: The discussion would be a good place to talk about this: pros and cons, comparing limitations of this approach vs. nonlinear mixed effects as a pipeline for generating individualized models**

AR: We believe that an extensive discussion of the various parameter estimation techniques is outside the scope of this manuscript. However, as mentioned above we fully acknowledge the applicability of other parameter estimation techniques for estimating parameters on the population and individual levels. Therefore, as recommended, we have included brief remarks discussing the potential of the other techniques (page 18, lines 359-366).

**RC 9: Principle component analysis: Does not necessarily reduce all parameters to two dimensions. Were additional dimensions examined? Did any other trends appear in comparisons of other PCs?**

AR: We have solely used PCA in order to visualize the parameter space. Clarifications have been added to the text on page 9, lines 177-179 and on page 15, lines 281-283 to emphasize this. While the other components were also examined, there was no interesting clustering of the data. The development of T2DM happens continuously over a prolonged period of time: as insulin resistance progresses, insulin secretion first compensates and later declines due to prolonged stress on the pancreatic beta islets. Subjects' responses here represent snapshots throughout this trajectory. Therefore, we believe that the lack of clustering and more of a spectrum along the loading directions is sensible. Furthermore, the relatively homogeneous population of "healthy overweight/obese" individuals also reinforces this notion.

**RC 10: Page 8/9: Need to specify the cut-offs used to remove non-physiological glucose or insulin responses. Were full subjects removed, or just datapoints outside the acceptable limits?**

AR: Full subjects were removed prior to the analyses due to a "non-physiological response" in case no glucose and no insulin response was observed in the OGTT profile (commonly referred to as a "failure of the OGTT" in clinical settings). The identification of these cases were aided by clinicians from the department of internal medicine. Clarification has been added to the text on page 9, line 188-190.

**RC 11: Table 1: please provide definitions for abbreviations in the footnote (NGT, IFG, IGT).**

AR: The requested information has been added to the footnote, as well as a reference to the Table S1, where the criteria for the abbreviations are elaborated.

**RC 12: Figure 4: What are the implications of the fact that the NGT group's parameter values span the range of all different groups? That an unusual value in a single parameter is not an indication of declining glucose tolerance or insulin production? This is interesting, and it would be nice to explore further: any correlations between parameters, etc. that appear in certain groups?**

AR: We believe that the primary implication is that it is insufficient to characterize these responses based on only baseline and/or 2h post-load measurements. Instead, the complete glucose-insulin dynamics should be evaluated in order to gain insight into the glucose homeostasis. We propose to use personalized mechanistic models to do this, where the model parameters contain information from the full dynamics and the interplay between glucose and insulin. In this "obese/overweight but otherwise healthy" population, the fasting and 2h post-load measurements are frequently borderline cases and quantifying the complete dynamics the model parameters may indicate that the response is indeed more similar to an insulin resistant-type or a type 2 diabetic response. We have included these remarks on page 20, lines 406-410, 423-429.

**RC 13: In the PCA, the ADA guideline-determined subgroups do not separate into different clusters. The authors say this highlights the limitations of these classifications. It would be nice to go a step further and show if the model does better in some way at determining which subjects are at highest risk of progressing to T2DM or similar. Similar to the last point, are there specific metrics or classifications that can be derived from the model that would be useful to clinicians and patients? Generally, if some such insight can be distilled into simpler metrics (e.g. subgroups, clusters, a ratio of two parameters) that will help the doctor make decisions about patient care, this substantially improves likelihood of clinical translation. The authors hint at**

**this in the discussion: the NGT group contains some individuals with high insulin in order to maintain normal glucose levels at 2 hours, suggesting insulin resistance. Could you show these groups, classified using the model, in Figure 5 (or another figure)? Do you need the model to derive these, or does the model largely suggest the value of also measuring insulin at 2 hours?**

AR: Indeed, while the ADA criteria capture a clinically relevant property it is slightly different to what the model captures. The ADA criteria are sufficient for clinical diagnosis of the prediabetes types and type 2 diabetes, however it fails to capture the nuances of the responses that may ultimately yield therapeutic implications. The model parameter for insulin uptake (k5) associated with the Matsuda index, a frequently used measure of insulin sensitivity. Furthermore, the association of the insulin secretion parameters (k6 and k8) with the insulinogeneic index has been evaluated as requested by the reviewers. Therefore, we provide some insight into what the model parameters might add to a more nuanced characterization of the individuals' glucose homeostasis on page 20, lines 406-410, 423-429. Future application of the model on studies with deeper phenotyping (e.g. hyperinsulinemic-euglycemic clamp) could allow further exploring the estimated model parameters.

The grouping of the high insulin NGT individuals is difficult due to the lack of a clinical/biological definition of high insulin values. Our comment on this was purely an observation and does not aim to derive cut-offs for intermediate time points of the OGTT. However, the parameter values directly relate to physiological processes which is also reinforced by the external measures (Matsuda, Insulinogenic index) in case of k5, k6 and k8. Therefore, the estimated parameter values themselves are valuable indicators of insulin sensitivity/insulin secretion, potential alternatives to the more simplistic or steady state approximations currently widely used in clinical settings, see page 20, lines 423-429.

**RC 14: Do you envision these models as a better way to generate cohorts of virtual patients with varying glucose metabolism? Do you feel that, with sufficient ranges of patients to fit to, this pipeline would also work well for subjects with early or late T2DM? What about T1DM?**

AR: We are grateful for the reviewer for pointing out the personalized models as a way to generate virtual patients. We agree that this is an extremely interesting potential, and have added some remarks to this on page 20, lines 427-429. The original aim of the underlying model used in this study was to help educate diabetes patients (both type 1 and type 2) to understand how certain perturbations (meals) modulate their postprandial glucose metabolism. However, this was only possible in a general way by playing with a virtual type 2 diabetic individuals' simulations. As the reviewer points out, using heterogeneous measured data, as described in the current study, from a sufficient range of patients is a crucial step towards being able to tune the model to an individual. We believe that with the appropriate data and the workflow described in this paper, the model may be tuned to a range of specialized T2DM conditions as well. The question of T1DM patients is interesting, since they lack internal insulin secretion and therefore the model must account for external insulin administration. The EDES model as described in Maas 2017 [ref 24] already included a way to account for external insulin, therefore we believe that this is indeed possible. However, for this population we disabled these parts of the model and exploring it further is outside the scope of this study.

**RC 15: Generally, it would be nice to clean up the spacing and labels on figures:**

**RC 15.1: While all are legible, spacing between subplots and titles is not always consistent (e.g. Figure 4).**

**RC 15.2: Axes labels are not all consistent (e.g. time marked at 60 and 120 min in Figure 3 (good), but only 0 and 100 in Figures 1 and 2).**

**RC 15.3: In places like Figure 4, where we are looking at parameter distributions by subgroup, it would be helpful to reference the physiological meaning of parameters in footnotes or subplot titles.**

**RC 15.4: In Figure 5, adding descriptive text into the figure to show which panels indicate e.g. increasing insulin sensitivity would make it much easier to digest the author's point here.**

AR: The figures have been revised to include the indicated improvements (comments 15.1-3). However, for sake of readability, we did not add more information to the figure 5 (figure 6 in the revised manuscript), as we believe that it already contains a substantial amount of information and further additions may decrease legibility.

**RC 16: I would suggest providing the supplemental figures as a more accessible format than .eps (pdf, tiff, etc): since supplemental files are not typically converted by journals, not all readers may have programs that can open .eps files, or spend the time to look at these figures.**

AR: We appreciate the reviewer pointing out our mistake. All supplemental figures are replaced by .tiff files.

February 10, 2021
