# Supplementary material for: Personalized computational model quantifies heterogeneity in postprandial responses to oral glucose challenge
Source: PLoS Comput Biol. 2021 Mar 31;17(3):e1008852. doi: 10.1371/journal.pcbi.1008852 (PMC8011733; doi:10.1371/journal.pcbi.1008852)
Supplement: Attachment — Submitted filename: PCB Nove 2020 Response.docx [file pcbi.1008852.s015.docx]

Erdos et. al. present a model aiming to better characterize the heterogeneity in individual responses to oral glucose challenges. The authors build “personalized models” for each individual by fitting a subset of parameters to each individual. Overall, I have no explicit concerns with the technical aspects of the work. However, the authors could do more to make the manuscript accessible to readers, to place their work in context of other nonlinear mixed effect modeling approaches, and to bridge towards how this work can be clinically translated.

Specific points:

- When the authors use words like “individualized models” and “model selection,” this generally implies that different model structures are being compared, or selected for each subject. I’d suggest wording like “personalized models” and “selection of parameters for personalized fitting” to make it clearer that all individuals get the same model structure but personal parameterizations.
- While Appendix S1 provides details on the model, it would be very helpful to include a figure schematic in the main text for readers to reference. For example, when looking at the list of parameters to be individualized, this would help the reading think about what processes k1, k4, k5, k6, k8, and k9 represent, compared to the processes for which parameters are not individualized.
- Dmeal (food intake): Clarify in Table S1 of appendix S1 that this is specifically glucose (since an OGTT, not all food intake).
- Given the population and individualization steps, and then the subgroups considered, it would be helpful to the readers to provide a flow chart of the model building process. From just the text, it is a bit hard to clearly understand the full process.
- In the methods or supplement, please lay out the ADA diagnosis criteria, for reference by unfamiliar readers.
- Parameter estimation step: was this fit to all data (naïve pooled) or mean values for the population?
- Model Individualization Pipeline: Why were non-individualized parameters set to literature values instead of the population values fit in the parameter estimation step?
- Can you discuss why you chose this approach over a “traditional” nonlinear mixed-effect modeling approach, which also lets you fit individual estimates for some parameters and fix other to population levels? I am not suggesting the authors change their methodology, as both approaches are reasonable, but I think this is worth discussing; a fair bit of the modeling to date on insulin-glucose dynamics (e.g. IGI model, which should also be referenced), and many members of the authors’ prospective audience here come from the pharmacology field, where many would expect to see this done in a nonlinear mixed-effect modeling approach. This is particularly relevant as the authors present this as a generalizable pipeline.
  - The authors do compare individual and population performance by looking at each of the patient subgroups. This verification is reassuring (It would be concerning if the individual models to not do better than population), and Figure 3, laying out some of the interesting individual fits and patterns is quite nice.
  - However, the authors could go farther in diagnosing individual and population model predictions, as well as visualizing distributions of individual parameters and residual errors. This would allow readers to get a feel for how well the model is performing, and whether certain assumptions one generally makes with this sort of modeling are being met. Figures 6 and 7 in this tutorial provide a good example of the type of diagnostic plots that would be helpful. They don’t need to be exactly the same (e.g. I don’t expect conditional weighted versions of residuals), but generally would help show if the individual and population components of the model are suitable and well-behaved: <https://ascpt.onlinelibrary.wiley.com/doi/full/10.1038/psp.2013.14>
  - The discussion would be a good place to talk about this: pros and cons, comparing limitations of this approach vs. nonlinear mixed effects as a pipeline for generating individualized models.
- Principle component analysis: Does not necessarily reduce all parameters to two dimensions. Were additional dimensions examined? Did any other trends appear in comparisons of other PCs?
- Page 8/9: Need to specify the cut-offs used to remove non-physiological glucose or insulin responses. Were full subjects removed, or just datapoints outside the acceptable limits?
- Table 1: please provide definitions for abbreviations in the footnote (NGT, IFG, IGT).
- Figure 4: What are the implications of the fact that the NGT group’s parameter values span the range of all different groups? That an unusual value in a single parameter is not an indication of declining glucose tolerance or insulin production? This is interesting, and it would be nice to explore further: any correlations between parameters, etc. that appear in certain groups?
- In the PCA, the ADA guideline-determined subgroups do not separate into different clusters. The authors say this highlights the limitations of these classifications. It would be nice to go a step further and show if the model does better in some way at determining which subjects are at highest risk of progressing to T2DM or similar. Similar to the last point, are there specific metrics or classifications that can be derived from the model that would be useful to clinicians and patients? Generally, if some such insight can be distilled into simpler metrics (e.g. subgroups, clusters, a ratio of two parameters) that will help the doctor make decisions about patient care, this substantially improves likelihood of clinical translation. The authors hint at this in the discussion: the NGT group contains some individuals with high insulin in order to maintain normal glucose levels at 2 hours, suggesting insulin resistance. Could you show these groups, classified using the model, in Figure 5 (or another figure)? Do you need the model to derive these, or does the model largely suggest the value of also measuring insulin at 2 hours?
- Do you envision these models as a better way to generate cohorts of virtual patients with varying glucose metabolism? Do you feel that, with sufficient ranges of patients to fit to, this pipeline would also work well for subjects with early or late T2DM? What about T1DM?
- Generally, it would be nice to clean up the spacing and labels on figures:
  - While all are legible, spacing between subplots and titles is not always consistent (e.g. Figure 4).
  - Axes labels are not all consistent (e.g. time marked at 60 and 120 min in Figure 3 (good), but only 0 and 100 in Figures 1 and 2).
  - In places like Figure 4, where we are looking at parameter distributions by subgroup, it would be helpful to reference the physiological meaning of parameters in footnotes or subplot titles.
  - In Figure 5, adding descriptive text into the figure to show which panels indicate e.g. increasing insulin sensitivity would make it much easier to digest the author’s point here.
- I would suggest providing the supplemental figures as a more accessible format than .eps (pdf, tiff, etc): since supplemental files are not typically converted by journals, not all readers may have programs that can open .eps files, or spend the time to look at these figures.
